# The price of personal mobility: burden of injury and mortality from personal mobility devices in Singapore - a nationwide cohort study

**DOI:** 10.1186/s12889-019-7210-6

**Published:** 2019-07-04

**Authors:** Aidan Lyanzhiang Tan, Trauma Coordinators and Trauma Service Representatives, Chee Keong Chong, Haslizah Bte Hassan, Serena Koh, Yan Li, Kebing Lin, Nadhirah Binte Sani, Peifu Ng, Christelle Cha Sow King, Tiong Thye Goo, Woan Wui Lim, Michael Liu, Sanjay Patel, Hong Chuen Toh, Suat Ting Lim, Tian Zhi Lim, Lynette Mee Ann Loo, Sabrina Siok Buay Yeo, Antony Gardner, Kai Yin Hwang, Noor’ Aini Binte Nordin, Yee Kent Liew, Jen Heng Pek, Pei Leng Chong, Norhayati Mohamed Jainodin, Seo Kiat Goh, Chung Fai Jeremy Ng, Teng Teng Fiona Peh, Mann Hong Tan, Choon Peng Jeremy Wee, Yew Lok Woo, Jolene Y. X. Cheng, Karen Tsung Shyen Go, Serene S. N. Goh, Xin Yi Leong, Li-Tserng Teo, Nivedita Nadkarni, Ting Hway Wong

**Affiliations:** 1 Preventive Medicine, National University Hospital, 1E Kent Ridge Road, NUHS Tower Block, Level 12, Singapore, 119228 Singapore; 2 Health Services Research Unit, Singapore General Hospital, 226 Outram Road, 169039 Singapore, Singapore; 3 Centre for Quantitative Medicine, Duke-National University of Singapore Graduate Medical School, 8 College Rd, Singapore, 169857 Singapore; 4 Singapore General Hospital/Duke-National University of Singapore Graduate Medical School, Outram Road, Singapore, 169608 Singapore; 5 Department of General Surgery, Singapore General Hospital, Outram Road, Singapore, 169608 Republic of Singapore

**Keywords:** Trauma, Personal mobility devices, E-scooters, Injury severity, Injury prevention, Singapore, Asia

## Abstract

**Background:**

Personal mobility devices (PMDs) like skate-scooters, electric bicycles (e-bikes) or motorised scooters (e-scooters) have become widely available globally. There are several studies describing the rising incidence of injury from such devices. The aim of our study was to examine PMD user factors between motorised (MotPMDs) vs non-motorised PMDs (NonPMDs) as risk factors for severe injury and the need for hospital admission.

**Methods:**

We analysed de-identified National Trauma Registry data (2015 to 2017) from all public sector hospitals in Singapore for patients aged 12 and above presenting to emergency departments with PMD-related injuries. Multivariable logistic regression was used to identify risk factors for the primary outcome of interest (higher injury severity, defined as Injury Severity Score / ISS > =9), and the secondary outcome of interest (need for hospital admission). Additional subgroup analysis was conducted comparing only scooters (manual vs electric), the most common sub-type of PMD in our study.

**Results:**

Of the 614 patients in our study, majority were male (74%), median age 33 years, with 136 (22%) sustaining injuries with ISS > =9; 185 (30%) admitted [median stay length 3 days (IQR: 1–6)] and 93 (15%) required surgery. MotPMDs were more common (480, 78%), with e-scooters being the most common motorised device (393, 64%). There were 6 deaths, all in MotPMD users.

On both univariate and multivariable regression, MotPMD users [OR 3.82, 95% CI 1.51–12.9, *p* = 0.01] and older users (> = 60 years) [OR 9.47, 95% CI 2.45–62.9, *p* = 0.004] were more likely to sustain injuries with ISS > =9, and more likely to need admission (MotPMD users [OR 1.8, 95% CI 1.04–3.29, *p* = 0.045], age > =60 years [OR 4.72, 95% CI 1.86–13.0, *p* = 0.002]).

**Conclusion:**

MotPMDs tripled the risk of severe injury and doubled the risk of requiring hospitalisation, compared to NonPMDs, likely due to higher travelling speeds. Increased age was also associated with severe injury and requiring hospitalisation.

## Background

Personal mobility devices (PMDs), such as skate-scooters, and motorized personal mobility devices such as electric bicycles (e-bikes) or motorised scooters (e-scooters) have rapidly increased in popularity globally. There has been a corresponding rise in PMD-related injury [1–5], with unusual injury patterns being described [1, 6], particularly with hoverboards [7–11].

Motorised PMD (MotPMD) users, in particular, appear to be at greater risk of injury [5, 12, 13]. Reasons proposed include greater risk-taking behaviour for MotPMD users compared to non-motorised PMD users (NonPMDs) [14–17], and environmental separation issues (pedestrian vs bicycle lanes vs roads) [18]. MotPMDs also travel at much higher speeds than NonPMD [4, 17, 19]. While there appears to be a higher risk of injury for MotPMD users [5], there are very few studies evaluating the relationship between PMD type (MotPMD vs NonPMD) and the severity of injury. Existing studies have so far only evaluated e-bikes in relation to conventional bicycles, but other MotPMD types such as e-scooters are becoming more popular [12, 13].

In Singapore, trauma is the leading cause for hospitalisation, and one of the major causes of mortality [20]. The Singapore National Trauma Registry (NTR) was established in 2011, covering all public hospitals in Singapore. Using NTR information, our primary goal was to identify and evaluate risk factors for higher injury severity (Injury Severity Score, ISS > =9) [21, 22], and for inpatient hospital admission in PMD users.

## Methods

### Data source and data collection

This retrospective national cohort study was conducted using NTR data from all adult public-sector hospitals in Singapore. The NTR covers all public hospitals in Singapore, with coding and data collection conducted by trained trauma data coordinators with annual data quality checks (covering accuracy, reliability, completeness and validation) performed annually. Quarterly review of data capture is performed by the National Registry of Diseases Office. The registry inclusion criteria, data collection, data cleaning and data quality audit processes have been described in previous studies [23–25].

### Study design

Retrospective data from January 2015 to December 2017 was extracted and de-identified by the public hospital trauma units, with 1 site contributing data only for 2015–2016, and another site providing only 2016 data. The associations between patient demographics, PMD types (MotPMD vs NonPMD), and outcomes of interest (injury severity and need for hospitalization) were examined.

### Study population

We included patients aged 12 years and older who presented at the public-sector hospital emergency departments with PMD-related injuries. We excluded injuries related to: motorised wheelchairs, roller blades, and bicycles. Although pedestrians injured by PMDs would contribute to the burden of injury by PMDs, the data collection definitions for pedestrians injured by PMDs had not been standardized across the sites at the time of this study, as the NTR categories had focussed on PMD riders, hence we excluded pedestrians injured by PMDs.

### Outcome measures

The primary outcome measure was higher injury severity, defined as injuries with an ISS of 9 or higher [21, 22].

The second outcome measure was the need for hospital admission, as a proxy for healthcare utilization and costs.

### Covariates

Patient demographics, PMD details, need for admission, and whether surgery was performed, were extracted from the NTR. Age was analyzed in these age-bands: 12–19 years, 20 to 39 years, 40 to 59 years, and 60 years or older.

PMDs were grouped into the following categories: MotPMDs (hoverboards/unicycles/e-wheels, motorised bicycles/e-bikes/powered assisted bicycles, motorised scooters/e-scooters, motorised skateboards), and NonPMDs (non-motorised scooters – including kick scooters or self-balancing scooters, and skateboards). PMD rider position was collected where available, where the patient was either the main rider, or a passenger/pillion rider on the PMD. For sites where information on injury prevention protective gear was collected, helmet use was examined.

### Statistical testing

Overall patient characteristics were summarized by mean (standard deviation), median (inter-quartile range), or frequency (%) as appropriate.

Linear predictors of interest were re-categorised (for example, age into age-bands) for analysis. Logistic regression was used to examine the association between the variables and outcomes of interest.

Variables identified as having statistically significant associations (*p* < 0.05) in the univariate regression were included in the multivariable logistic regression model. Variables not statistically significant but clinically meaningful (gender) were also included.

As the morphology of injury due to different PMD types may differ, additional subgroup analysis was conducted of scooters only, comparing electric and non-electric scooters.

R version 3.4.3 was used.

### Ethical issues

Ethical approval by the corresponding author’s institution’s institutional review board was granted, with waiver of informed consent granted as only secondary retrospective and de-identified data was used.

## Results

### Descriptive analysis

We identified 732 potentially eligible patients from the registry and excluded 12 as being below 12 years old. The following patients were excluded for not meeting the criteria of a PMD rider: 89 (pedal cycles), 12 (roller blades) and 1 (motorised wheelchair), and 4 pedestrians injured by PMDs. Of the remaining 614 patients, majority were main riders (*n* = 600, 97%), the remainder were pillion riders. Users with undocumented PMD type (*n* = 42) were included in the overall descriptive analysis (of 614 patients) but excluded from the comparison of MotPMD vs NonPMD (572 patients).

For the 614 PMD riders, the median age was 33 years old (IQR: 24–46 years). Males accounted for 76% of the sample (*n* = 465). The predominant type of MotPMD used was motorised scooters/e-scooters (*n* = 393, 64%), followed by motorised bicycles/e-bikes/powered assisted bicycles (*n* = 63, 10%). The most common NonPMD were scooters (*n* = 47, 8%) and skateboards (*n* = 45, 7%). For 131 patients, use of injury prevention protective gear was documented, and only a minority wore helmets (*n* = 18, 14%).

Median ISS was 2 (IQR: 1–5), with almost a quarter sustaining significant injury (ISS > =9) (*n* = 136, 22%), and more than a quarter of patients requiring admission (*n* = 185, 30%). The average length of stay was 3 days (IQR 2–6). Only 1 in 6 patients required surgical intervention (*n* = 93, 15%). Of the 614 patients, 6 died (0.97%); 3 aged between 40 and 59 years, and 3 aged more than 60 years of age. All 6 deaths were in MotPMD users (Table 1).

**Table 1:** PMD riders descriptive statistics

		Overall		Non-motorised PMD users		Motorised PMD users	
		*Number (%) / Median (IQR) / Mean (SD)*					
Total		614		92		480	
Age (years)^†^	12 to 19	48	(8%)	18	(20%)	25	(5%)
	20 to 39	347	(57%)	56	(61%)	267	(56%)
	40 to 59	168	(27%)	16	(17%)	141	(29%)
	> = 60	51	(8%)	2	(2%)	47	(10%)
Gender	Females	149	(24%)	20	(22%)	111	(23%)
Personal Mobility Device (PMD) Type	Motorised scooter / e-scooter	393	(64%)	–		393	(82%)
	Motorised bicycle / ebike / powered assisted bicycle	63	(10%)	–		63	(13%)
	Hoverboards / unicycle / e-wheel	22	(4%)	–		22	(5%)
	Motorised skateboard	2	(0.30%)	–		2	(0.45%)
	Scooter - kickscooter, self balancing	47	(8%)	47	(51%)	–	
	Skateboard	45	(7%)	45	(49%)	–	
	Undocumented PMD type	42	(7%)	–		–	
Protective Gear Use	Helmet used (data available for 131 riders)	18	(14%)	0	(0%)	17	(4%)
Riding Position	Main Rider	600	(98%)	92	(100%)	469	(98%)
	Pillion / Passenger	14	(2%)	0	(0%)	11	(2%)
Injury Details^†^	ISS > = 9	136	(22%)	4	(4%)	90	(19%)
Treatment Details	Admitted^†^	185	(30%)	17	(18%)	159	(33%)
	Average Length of Stay (days)	3	(2–6)	3	(2–6)	3	(1–6)
	Required Surgery	93	(15%)	10	(11%)	81	(17%)
Deaths		6	(0.97%)	0	(0%)	6	(1.25%)

^†^Statistically significant (*p* < 0.05) difference between Motorised and Non-Motorised PMD users

Riders of MotPMDs tended to be older (median age 34 vs 25 years for non-motorized, *p* < 0.01). Gender distribution was similar between the two groups. More MotPMD users had significant injuries (19% vs 4%, *p* < 0.01), and more required admission (33% vs 18%, *p* < 0.01). The proportion requiring surgery was similar between the groups (Table 1).

### Univariate Analysis

MotPMD users were more likely to sustain significant injury (ISS > =9)(OR 5.08, 95% CI 2.05–16.9, *p* = 0.002). Older age was also associated with sustaining higher injury severity (age-group 60 years and above, OR 4.03, 95% CI 1.59–11.28, *p* < 0.01). MotPMDs (OR 2.16, 95% CI 1.26–3.90, *p* = 0.007) and the older age group (OR 4.75, 95% CI 2.02–11.9, *p* = 0.001) were both associated with increased likelihood of hospital admission (Table 2).

**Table 2:** Factors associated with severe injury or need for admission (*n* = 572)

Outcome	Variables	Univariate			Multivariable		
		*Odds Ratio, (95% CI), p value*			*Odds Ratio, (95% CI), p value*		
Severe Injury	Motorised PMD (ref non-motorised)	5.08	(2.05, 16.9)	< 0.01	3.82	(1.51, 12.9)	0.01
	12–19 years (ref)						
	20–39 years	1.24	(0.56, 3.15)	0.61	2.26	(0.64, 14.3)	0.28
	40–59 years	2.21	(0.97, 5.69)	0.07	4.88	(1.36, 31.2)	0.04
	> =60 years	4.03	(1.59, 11.28)	< 0.01	9.47	(2.45, 62.90)	< 0.01
	Gender (ref female)	1.08	(0.70, 1.70)	0.74	1.82	(1.00, 3.51)	0.05
Admission	Motorised PMD (ref non-motorised)	2.16	(1.26, 3.90)	< 0.01	1.80	(1.04, 3.29)	0.04
	12–19 years (ref)						
	20–39 years	1.31	(0.65, 2.87)	0.48	1.38	(0.63, 3.34)	0.45
	40–59 years	1.85	(0.89, 4.17)	0.12	2.03	(0.90, 5.07)	0.10
	> =60 years	4.75	(2.02, 11.88)	< 0.01	4.72	(1.86, 13.03)	< 0.01
	Gender (ref female)	1.37	(0.91, 2.09)	0.14	1.23	(0.79, 1.94)	0.36

### Multivariable analysis

MotPMD use (OR 3.82, 95% CI 1.51–12.9, *p* = 0.01), male gender (OR 1.82, 95% CI 1.00–3.51, *p* = 0.06) and the older age groups (40–59 years, OR 4.88, 95% CI 1.36–31.2, *p* = 0.04, and > = 60 years, OR 9.47, 95% CI 2.45–62.9, *p* < 0.01) were significantly associated with sustaining severe injury (ISS > =9).

Only MotPMD use (OR 1.80, 95% CI 1.04–3.29, *p* = 0.045) and the older age group (> = 60 years) (OR 4.72, 95% CI 1.86–13.0, *p* = 0.002) were significantly associated with hospital admission on multivariable analysis (Table 2). Male gender showed a slightly higher risk of admission, but this was not statistically significant (OR1.23, 95% CI 0.79–1.94, *p* = 0.36).

### Subgroup analysis – E-scooter vs non-motorised scooters

We compared 393 e-scooter users against 47 non-motorised scooter users. Age > =60 years (OR 6.0, 95% CI 0.99–115, *p* = 0.10), male gender (OR 2.24, 95% CI 1.07–5.30, *p* = 0.04) and MotPMD use (OR 3.78, 95% CI 1.11–23.7, *p* = 0.07) were associated with sustaining significant injury, although age fell short of significance on multivariable analysis (Table 3).

**Table 3:** Factors associated with severe injury or admission: scooters vs e-scooters subgroup (*n* = 440)

Outcome	Variables	Univariate			Multivariable		
		*Odds Ratio, (95% CI), p value*			*Odds Ratio, (95% CI), p value*		
Severe Injury	Motorised PMD (ref non-motorised)	3.97	(1.18, 24.8)	< 0.01	3.78	(1.11, 23.65)	< 0.01
	12–19 years (ref)						
	20–39 years	2.43	(0.48, 44.50)	0.39	2.51	(0.49, 46.17)	0.38
	40–59 years	4.51	(0.87, 83.00)	0.15	4.74	(0.9, 87.72)	0.14
	> =60 years	6.67	(1.12, 128.00)	< 0.01	6.00	(0.99, 115.78)	0.10
	Gender (ref female)	2.31	(1.12, 5.41)	0.03	2.24	(1.07, 5.30)	0.04
Admission	Motorised PMD (ref non-motorised)	1.28	(0.66, 2.65)	0.48	1.25	(0.64, 2.59)	0.54
	12–19 years (ref)						
	20–39 years	0.89	(0.35, 2.59)	0.83	0.91	(0.35, 2.66)	0.86
	40–59 years	1.22	(0.46, 3.63)	0.70	1.25	(0.47, 3.73)	0.67
	> =60 years	2.24	(0.73, 7.49)	0.17	2.18	(0.71, 7.34)	0.19
	Gender (ref female)	1.34	(0.83, 2.23)	0.24	1.27	(0.78, 2.13)	0.34

## Discussion

To our knowledge, our study is the first to examine factors associated with injury severity and hospitalization in personal mobility device riders across a range of PMD types, using data that is nationally representative in an urban city state. Other studies have focused on specific types of PMDs. We found that MotPMD users had higher injury severity scores, with a greater proportion requiring surgical intervention compared to NonPMD users, as has been reported by other authors [5, 18]. Our study, however, is one of the few that included a NonPMD comparator group, suggesting that MotPMD users are at higher risk of injury compared to NonPMD users.

MotPMD use and older age were identified as factors independently associated with severe injury. A similar pattern was found in the subgroup analysis of e-scooters, although this only reached statistical significance for severe injury, possibly due to smaller subgroup sample size. This is in keeping with the findings from studies conducted in China and Switzerland [3, 13], focusing on pedal cycles versus electric-bicycle users, which also found higher injury severity for electric-bicycle users.

Older age was associated with higher risk of significant injury or admission, likely due to higher fall risk and frailty. Studies of motor vehicle crashes have also shown that the older patients tend to sustain higher injury severity and mortality [25–29]. In common with motor vehicle crash-injured patients, older PMD riders are likely to have reduced physiological reserves and impaired response to injury [30–32], coupled with comorbid conditions that predispose to injury or further reduce resilience [32, 33], and propensity for fracture even with minor trauma [34]. In addition, older riders are probably choosing to ride MotPMDs to overcome their pre-existing musculoskeletal problems, and these patients may be more frail than younger patients to begin with.

While our study did not specifically examine PMD rider behaviour, we did find that the majority of the MotPMD users did not use protective helmets, in line with the risk-taking profile found in other studies [3, 13–17, 28].

MotPMD-related reasons such as higher travelling speeds [4, 17, 19], and environmental issues such as lack of dedicated lanes vs usage of mixed-use conventional bicycle lanes, sidewalks or roads have also been proposed as contributory factors to injury [18]. MotPMD-related legislation varies between countries. In the European Union, MotPMDs are barred from motor vehicle roads, and are allowed only on sidewalks at speeds < 6 km/hr. [35]. In the United States, there is great variability between the states, with some permitting road usage [35]. In Singapore, new legislation (Active Mobility Act) now restricts use to only sidewalks or shared bicycle lanes, but at maximum speed of 25 km/hr. Our study was conducted prior to the implementation of the new regulations. Future studies focusing on interventional strategies for injury prevention would be of great policy interest, such as examining the differences in incidence and economic burden of PMD-related injury before and after legislative rules controlling MotPMD use, especially given the significant association of MotPMDs with severe injury and admissions.

The main strength of our study was that it covered all the public-sector institutions nation-wide in an urban multi-ethnic population. However, the use of NTR data had some limitations. Certain information such as PMD type and helmet use was incomplete. Nevertheless, data quality was good for injury severity and need for hospital admission, data that is routinely collected by the NTR. Another limitation is that the NTR only captures data for patients who present to the emergency department of public hospitals, and hence our study findings may not apply to riders who self-medicate or see their family physician.

## Conclusion

MotPMD riders had triple the risk of severe injury and double the risk of requiring hospitalisation compared to NonPMDs. Increased age was also associated with severe injury and requiring hospitalisation.

## Data Availability

The de-identified data from this study was obtained from the respective trauma offices of the participating hospitals, after permissions were sought and granted from the respective trauma office directors upon proof of ethical approval. This data is also submitted to the National Trauma Registry, established and funded by Singapore’s Ministry of Health. Pooled and de-identified data may be available from the National Registry of Diseases Office in Singapore for researchers who meet the criteria for access to confidential data. Details are available at https://www.nrdo.gov.sg/faq.

## Abbreviations

ISS: Injury Severity Score
MotPMD: Motorised Personal Mobility Device
NonPMD: Non-motorised Personal Mobility Device
NTR: National Trauma Registry
PMD: Personal Mobility Device

## References

[1] Papoutsi S, Martinolli L, Braun CT, Exadaktylos AK (2014). E-bike injuries: experience from an urban emergency department-a retrospective study from Switzerland. Emergency medicine international.

[2] Du W, Yang J, Powis B, Zheng X, Ozanne-Smith J, Bilston L, He J, Ma T, Wang X, Wu M (2014). Epidemiological profile of hospitalised injuries among electric bicycle riders admitted to a rural hospital in Suzhou: a cross-sectional study. Inj Prev.

[3] Weber T, Scaramuzza G, Schmitt KU (2014). Evaluation of e-bike accidents in Switzerland. Accid Anal Prev.

[4] Zhang X, Yang Y, Yang J, Hu J, Li Y, Wu M, Stallones L, Xiang H (2018). Road traffic injuries among riders of electric bike/electric moped in southern China. Traffic injury prevention.

[5] Zhou SA, Ho AFW, Ong MEH, Liu N, Pek PP, Wang YQ, Jin T, Yan GZ, Han NN, Li G (2017). Electric bicycle-related injuries presenting to a provincial hospital in China: a retrospective study. Medicine.

[6] Tenenbaum S, Weltsch D, Bariteau JT, Givon A, Peleg K, Thein R, Group IT (2017). Orthopaedic injuries among electric bicycle users. Injury.

[7] Al-Kashmiri Ammar, Hasan Ahmed Q., Al-Shaqsi Sultan (2017). Rolling danger: the epidemiology of injuries caused by hover boards in the United States in five years (2011–2016). Journal of Emergency and Critical Care Medicine.

[8] McIlvain Cody, Hadiza Galadima, Tzavaras Theodore J., Weingart Gregory S. (2019). Injuries associated with hoverboard use: A review of the National Electronic Injury Surveillance System. The American Journal of Emergency Medicine.

[9] Schapiro AH, Lall NU, Anton CG, Trout AT (2017). Hoverboards: spectrum of injury and association with an uncommon fracture. Pediatr Radiol.

[10] Siracuse BL, Ippolito JA, Gibson PD, Beebe KS (2017). Hoverboards: a new cause of pediatric morbidity. Injury.

[11] Sobel AD, Reid DB, Blood TD, Daniels AH, Cruz AI (2017). Pediatric orthopedic hoverboard injuries: a prospectively enrolled cohort. J Pediatr.

[12] Gross I, Weiss DJ, Eliasi E, Bala M, Hashavya S (2018). E-bike–related trauma in children and adults. J Emerg Med.

[13] Hu F, Lv D, Zhu J, Fang J (2014). Related risk factors for injury severity of e-bike and bicycle crashes in Hefei. Traffic injury prevention.

[14] Bai L, Liu P, Guo Y, Yu H (2015). Comparative analysis of risky behaviors of electric bicycles at signalized intersections. Traffic injury prevention.

[15] Yang J, Hu Y, Du W, Powis B, Ozanne-Smith J, Liao Y, Li N, Wu M (2014). Unsafe riding practice among electric bikers in Suzhou, China: an observational study. BMJ Open.

[16] Wu C, Yao L, Zhang K (2012). The red-light running behavior of electric bike riders and cyclists at urban intersections in China: an observational study. Accid Anal Prev.

[17] Haustein S, Møller M (2016). E-bike safety: individual-level factors and incident characteristics. J Transp Health.

[18] Siman-Tov M, Radomislensky I, Peleg K, Group IT (2017). The casualties from electric bike and motorized scooter road accidents. Traffic injury prevention.

[19] Cherry CR, Yang H, Jones LR, He M (2016). Dynamics of electric bike ownership and use in Kunming, China. Transport Policy.

[20] Singapore Health Facts [https://www.moh.gov.sg/resources-statistics]. Accessed 24 Nov 2018.

[21] Baker SP, O'neill B (1976). The injury severity score: an update. J Trauma Acute Care Surg.

[22] O'Reilly GM, Gabbe B, Cameron PA (2015). Trauma registry methodology: a survey of trauma registry custodians to determine current approaches. Injury.

[23] Wong Ting Hway, Nguyen Hai V., Chiu Ming Terk, Chow Khuan Yew, Ong Marcus Eng Hock, Lim Gek Hsiang, Nadkarni Nivedita Vikas, Bautista Dianne Carrol Tan, Cheng Jolene Yu Xuan, Loo Lynette Mee Ann, Seow Dennis Chuen Chai (2015). The Low Fall as a Surrogate Marker of Frailty Predicts Long-Term Mortality in Older Trauma Patients. PLOS ONE.

[24] Wong TH, Krishnaswamy G, Nadkarni NV, Nguyen HV, Lim GH, Bautista DCT, Chiu MT, Chow KY, Ong MEH (2016). Combining the new injury severity score with an anatomical polytrauma injury variable predicts mortality better than the new injury severity score and the injury severity score: a retrospective cohort study. Scandinavian Journal of Trauma, Resuscitation and Emergency Medicine.

[25] Wong TH, Nadkarni NV, Nguyen HV, Lim GH, Matchar DB, Seow DCC, King NKK, Ong MEH (2018). One-year and three-year mortality prediction in adult major blunt trauma survivors: a National Retrospective Cohort Analysis. Scand J Trauma Resusc Emerg Med.

[26] Sammy I, Lecky F, Sutton A, Leaviss J, O'Cathain A (2016). Factors affecting mortality in older trauma patients-a systematic review and meta-analysis. Injury.

[27] Beck B, Cameron P, Lowthian J, Fitzgerald M, Judson R, Gabbe BJ (2018). Major trauma in older persons. BJS open.

[28] Hsieh CH, Liu HT, Hsu SY, Hsieh HY, Chen YC (2017). Motorcycle-related hospitalizations of the elderly. Biom J.

[29] Hashmi A, Ibrahim-Zada I, Rhee P, Aziz H, Fain MJ, Friese RS, Joseph B (2014). Predictors of mortality in geriatric trauma patients: a systematic review and meta-analysis. The journal of trauma and acute care surgery.

[30] Frankenfield D, Cooney RN, Smith JS, Rowe WA (2000). Age-related differences in the metabolic response to injury. J Trauma.

[31] Banks SE, Lewis MC (2013). Trauma in the elderly: considerations for anesthetic management. Anesthesiol Clin.

[32] Bonne S, Schuerer DJ (2013). Trauma in the older adult: epidemiology and evolving geriatric trauma principles. Clin Geriatr Med.

[33] Llompart-Pou JA, Perez-Barcena J, Chico-Fernandez M, Sanchez-Casado M, Raurich JM (2017). Severe trauma in the geriatric population. World journal of critical care medicine.

[34] Reske-Nielsen C, Medzon R (2016). Geriatric Trauma. Emerg Med Clin North Am.

[35] Litman T, Blair R: Managing Personal Mobility Devices (PMDs) On Nonmotorized Facilities; 2019.

